# Predicting the Development of Type 2 Diabetes

**DOI:** 10.1371/journal.pmed.0020406

**Published:** 2005-11-01

Type 2 diabetes has been loosely defined as “adult onset” diabetes, but as diabetes becomes more common, cases are being diagnosed in younger people and children. In determining the risk of developing diabetes, environmental factors, such as food intake and exercise, are known to have an important role; most people with type 2 diabetes are either overweight or obese. Inherited factors are also important, but the genes involved remain poorly defined. In rare forms of diabetes, mutations of one gene can result in disease, whereas in type 2 diabetes, many genes are thought to be involved. One difficulty in understanding the genetic role is that genes associated with diabetes might show only a subtle variation in their sequence, and these variations may be extremely common. Hence, it can be very hard to link such common gene variations, known as single nucleotide polymorphisms (SNPs), with increased risk of developing diabetes.

One method of finding these diabetes genes is by whole-genome linkage studies in which associations between parts of the genome and risk of developing diabetes are looked for. Studies so far have identified several candidate genes associated with type 2 diabetes, although many results have been difficult to replicate. The list of genes for which there is good evidence from meta-analyses includes genes encoding for PPARG, calpain 10, Kir 6.2, and insulin receptor substrate-1 (IRS1).

These genes have a variety of effects; *PPARG* P12A polymorphism is associated with enhanced insulin sensitivity and protects against type 2 diabetes. Two SNPs in the gene encoding for cystein protease calpain 10 (CAPN10) confer increased susceptibility to insulin resistance and type 2 diabetes. Kir 6.2 is involved in glucose-stimulated insulin secretion in pancreatic cells. And carriers of a polymorphism in the *IRS1* gene have been shown to have reduced islet insulin content in pancreatic islets. [Fig pmed-0020406-g001]

**Figure pmed-0020406-g001:**
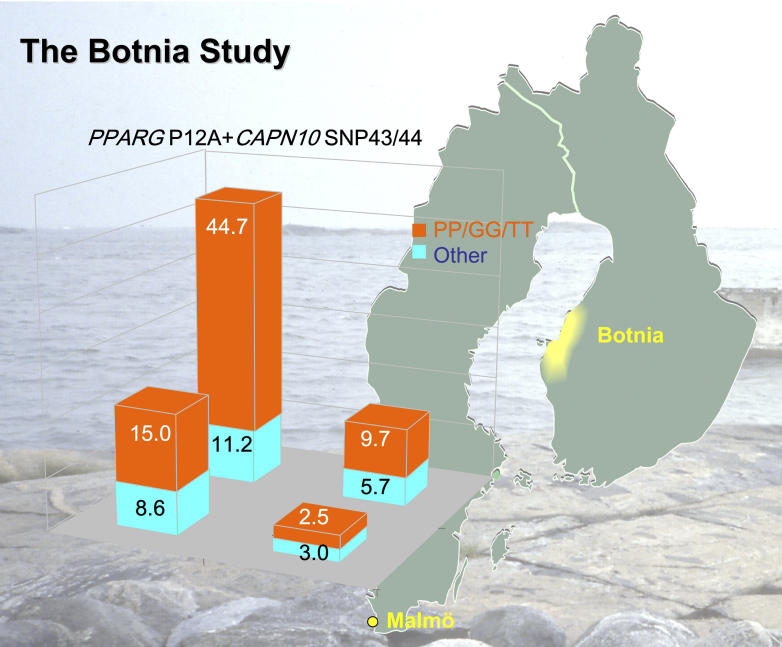
Genetic prediction of type 2 diabetes in the Botnia study

In this issue of *PLoS Medicine*, Valeriya Lyssenko and colleagues from Lund University sought to consolidate previous work by studying the predictive value of these variants for type 2 diabetes side by side in the largest study of its kind to date. They investigated the effect of these gene variants in 2,293 nondiabetic people aged 18–70 years old in western Finland—the Botnia study—over a median of six, range 2–12, years. In addition, they also studied the *uncoupling protein 2* gene (UCP2)—a polymorphism in the promoter of this gene (UCP2 −866G>A) (rs659366) has been associated in some, but not all, studies with increased risk of type 2 diabetes and impaired insulin secretion.

The study took place from 1990 to 2002, and enrolled patients from five health centers in western Finland who were asked to have health checks every two to three years. Six percent (132) of people developed type 2 diabetes. The key finding was that variants in the *PPARG* and *CAPN10* genes increased future risk for type 2 diabetes, particularly in individuals with other risk factors. In individuals with a high risk of developing diabetes—with a fasting plasma glucose (FPG) of 5.6 millimoles per liter and body mass index (BMI) of 30 kilograms per square meter—the hazard ratio increased to 21.2 for the combination of the *PPARG* PP and *CAPN10* SNP43/44 GG/TT genotypes compared with those with low-risk genotypes with normal FPG and BMI less than 30 kilograms per square meter.

The researchers found that replacing the family history with the *PPARG* and *CAPN10* variants in a predictive model (particularly in combination) gave almost the same strong prediction of subsequent type 2 diabetes. These genotypes also influenced the relationship between BMI and FPG, that is, in carriers of risk genotypes, there was a steeper increase in FPG for any given BMI.

The authors argue that the comparison of all the key gene variants side by side in one large study adds substantially to previous papers that have examined the effect of single gene variants on the risk of conversion to type 2 diabetes in interventional trials.

However, it is important to understand the effect of these variants on the risk of disease in a large, prospective observational study before studying additive or synergistic effects with interactions such as lifestyle changes, they said. One of the problems of other studies has been that results have been different between different subgroups.

Although this study has limited power, as the largest of its kind it suggests that genetic variants in candidate genes can predict future type 2 diabetes, particularly in association with conventional risk factors such as obesity and abnormal glucose tolerance. With accumulating data from prospective studies, it should be possible to define whether there will be a future role for genetic prediction of type 2 diabetes or whether these variants will influence response to prevention or treatment.

